# Acceptability and feasibility of using actigraphy to assess habitual physical activity and sleep parameters in men and women living in rural communities in conflict-affected Eastern Democratic Republic of Congo

**DOI:** 10.1017/gmh.2020.25

**Published:** 2020-11-20

**Authors:** Lisa J. Wood, Mervyn Christian, Nancy Perrin, Alfred Backikenge Mirindi, Jean Heri Banywesize, Clovis Murhula Mitima, Arsene Kajabika Binkurhorwa, Eric Mitima Ntqali, Gisele Ntakwinja Mushengezi, Mitima Mpanano Remy, Nancy Glass

**Affiliations:** 1 William F. Connell School of Nursing, Boston College, Chestnut Hill, MA, USA; 2 Johns Hopkins Bloomberg School of Public Health, Baltimore, Maryland, USA; 3 Johns Hopkins School of Nursing, Baltimore, Maryland, USA; 4 Programme d'Appui aux Initiative Economiques (PAIDEK) and Promotion de la Famille Paysanne (PFP), Bukavu, Democratic Republic of Congo

## Abstract

**Background:**

The goals of this study were to (1) determine the feasibility and acceptability of using actigraphy to objectively measure sleep quality and habitual physical activity in rural Democratic Republic of Congo (DRC) and (2) examine the relationship between sleep parameters, self-report symptoms, daytime physical activity, and physical function, including the ability to work.

**Method:**

Thirty individuals were asked to wear a wrist-worn accelerometer for 5 nights and 4 days. Nighttime sleep parameters derived were average and intra-individual variability (IIV) in total sleep time (TST), sleep onset latency (SOL), sleep efficiency (SE), and wake after sleep onset (WASO). Daytime habitual physical data derived were average and peak activity and daytime napping.

**Results:**

Ninety-three percent (*n* = 28) of participants completed the study. All participants who wore the device marked sleep and wake cycles and periods of non-wear using the marker. Trauma-related symptoms were not associated with mean or IIV in TST, SE, SOL, or WASO (*p* > 0.01). Those with higher levels of bodily pain slept longer (*β* = 0.633, *p* = 0.003, adjusted *R*^2^ = 0.279), were more likely to report that their physical health limited their physical activities (*β* = 0.71, *p* < 0.001, adjusted *R*^2^ = 0.679) and had greater difficulty doing daily work (*β* = 0.84, *p* = 0.001, adjusted *R*^2^ = 0.665).

**Conclusion:**

The use of actigraphy to collect objective measures of activity and sleep quality in rural post-conflict settings is feasible and acceptable. Our preliminary findings suggest that bodily pain and not trauma-related symptoms have a significant impact on sleep and functional outcomes in men and women survivors of prolonged conflict in the DRC.

## Background

In the Eastern Democratic Republic of Congo (DRC), rural populations have experienced more than two decades of conflict, political instability, and extreme poverty (Coghlan *et al*., 2006; Wakabi, 2008; Johnson *et al*., 2010). The situation in Eastern DRC remains volatile, with over 70 armed groups active in North and South Kivu provinces with limited access to protection, health care, education, and employment opportunities. Violence against civilians and misinformation campaigns continues in the region and is used as a ‘deliberate and strategic tactic’ (Omba Kalonda, 2008) to create fear and displacement, providing an opportunity for armed groups and authorities to pillage land and livestock in rural communities.

As a result of the economic deprivation, mental health is a significant public health issue for conflict-affected populations, such as in Eastern DRC. People experiencing poor mental health suffer substantial distress and may be more vulnerable to further violence, suicidality, and poor physical health and harmful health practices such as alcohol and drug abuse. Evidence has consistently demonstrated elevated rates of mental distress, most commonly reported as symptoms of post-traumatic stress disorder (PTSD) and depression, amongst diverse adult populations that have experienced conflict and internal displacement (Mollica *et al*., 1993; Roberts *et al*., 2009; Mugisha *et al*., 2015; Vonnahme *et al*., 2015; Cherewick *et al*., 2016). For example, in Uganda, PTSD, and depression amongst internally displaced persons have ranged between 44.5% and 75.3% (Mugisha *et al*., 2015). Factors that may affect mental health outcomes include gender, exposure to traumatic events, the experience of forced displacement, poverty, living conditions, and limited access to essential goods and services, all complex challenges identified in Eastern DRC (Kohli *et al*., 2015; Mugisha *et al*., 2015; Silove *et al*., 2017; Wells *et al*., 2018).

In 2010, Programme d'Appui aux Initiatives Economiques (PAIDEK), a Congolese microfinance organization, and the Johns Hopkins School of Nursing (JHSON) joined in collaboration to improve the economic security, health, and safety of rural households and communities the South Kivu province of Eastern DRC (Glass *et al*., 2012). The partners designed, implemented, and evaluated the economic empowerment program, Pigs for Peace (PFP), a hybrid livestock microfinance/productive asset transfer program that has been described elsewhere (Kohli *et al*., 2017). In addition to economic empowerment, we have previously reported on the potential of the PFP livestock/animal assets to moderate mental health symptoms for women that had experienced multiple conflict-related traumatic events. Specifically, as the household livestock/animal assets increased, the impact of conflict-related traumatic events on symptoms consistent with PTSD and depression was reduced (Glass *et al*., 2014). Therefore, livestock/animal assets and programs to support household productivity and economic stability can provide an added benefit by reducing mental health symptoms in settings that have minimal infrastructure and capacity to provide mental healthcare (Glass *et al*., 2014).

In our previous PFP studies, we have used solely self-report measures for symptoms associated with PTSD, and individuals with symptoms consistent with PTSD frequently report difficulty falling or staying asleep (Glass *et al*., 2014). Wrist-worn actigraphy has been used to objectively measure sleep disruption in PTSD (Khawaja *et al*., 2014; Theal *et al*., 2019). Actigraphy is a validated objective measure of sleep/wake cycles, and sleep data collected in this manner is highly correlated to polysomnography (PSG), a laboratory-based, procedure that is the current gold standard for measuring sleep (Lichstein *et al*., 2006). Actigraphy has many benefits over PSG, including the ease of data collection, and the ability to collect sleep data across several days in a natural rather than laboratory setting. In addition, wrist-worn actigraphy provides a measure of habitual physical activity, that is comparable to that collected by hip-worn accelerometers (Dieu *et al*., 2017).

Despite these benefits, the collection of habitual physical activity and sleep data by actigraphy may prove problematic in resource-poor, rural settings. A handful of studies have demonstrated feasibility and acceptability of collecting sleep data using wrist-worn actigraphy in pre-industrial electric-free communities (Knutson, 2014; de la Iglesia *et al*., 2015; Yetish *et al*., 2015; Samson *et al*., 2017)

The primary purpose of this study was to use the PFP infrastructure to determine the acceptability and feasibility of using wrist-worn actigraphy to obtain an objective measure and assess habitual physical activity and sleep parameters in rural, resource-poor communities in conflict-affected Eastern DRC (Arain *et al*., 2010). Secondary goals of the study were to examine the relationship between sleep parameters and trauma-related symptoms, daytime physical activity, and physical function, including the ability to work.

## Methods

### Study design and participants

This study collected objectively measured habitual physical activity and sleep data by actigraphy over 5 nights and 4 days leveraged existing baseline data from the parent PFP effectiveness evaluation, ‘*A Microfinance Intervention to Improve Health of Trauma Survivors in the Democratic Republic of the Congo’ (Pigs for Peace) (R01MD006075).* The parent study design was a randomized community trial to determine the effectiveness of PFP on household and participant economic security (e.g. livestock/animal assets, reduced credit), subjective health, and mental health. The parent study design and methods are presented elsewhere (Glass *et al*., 2017). Women and men aged 16 years and older, who expressed an understanding of the PFP program (repaying pig asset loan with two piglets), permanent residents and responsible individuals in the household in one of the ten PFP participating villages, were eligible for the parent study. Men and women were ineligible if they were younger than 16 years of age, did not express understanding of the PFP program, were not permanent residents or responsible for the household in the ten villages under study. Only men and women randomized to parent study intervention group and self-reported difficulty sleeping during baseline data collection were eligible for this study. After identifying eligible men and women from the baseline data, the team randomly selected three eligible participants, one male and two females (where possible) from each of the ten villages. After selection, the research assistant working in each village confirmed participant availability (i.e. were not traveling outside the village).

### Data collection procedures

The parent and current study questionnaire were developed to measure subjective health, PTSD, anxiety, depression, economic security, energy, and bodily pain using validated research instruments and findings from this team's prior research in DRC (Glass *et al*., 2017). To address the logistical challenges of working in an extremely low-resource and conflict-affected setting, the team collected baseline data in two phases of five villages each between 21 May 2012 and 8 November 2012. Approximately 2 months after baseline assessment, the team assessed habitual physical activity and sleep quality using actigraphy (Actiwatch Score, Respironics Inc.) on which this study is based. Since study interviews were conducted when participants would be earning their daily income, compensation for their time (~60–90 min) was provided per local rates, ~US$1.50.

Participants were trained by the PFP team after providing informed verbal consent and agreeing to one-on-one training on how to wear the Actiwatch Score (AW-S, Respironics). All of the training occurred on a Monday so that the first night of data collection (Monday night) was the same for all participants. Participants were shown how to wear the AW-S around the wrist of the non-dominant hand so that it did not move around on the wrist. They were asked to wear the device at all times except when in contact with large amounts of water (i.e. bathing or doing the laundry) and to mark their sleep and their wake times, or when they took the watch off, by pressing an indicator on the AW-S. Participants were asked to wear the device for 5 nights (Monday through Friday) and 4 days (Tuesday through Friday). During the training, various questions were posed to the participants to gauge their understanding of the usage of the AW-S. Each training session lasted from 45 to 90 min, with the longest training session lasting 180 min. Follow-up was conducted with participants the next day to address any issues that the participants may be facing. PFP staff collected the AW-S from each participant on Saturday.

### Demographics, household wealth, and food security

Demographic and information collected on the baseline survey of the PFP effectiveness study and reported for this study included age, marital status, and perceived household wealth in comparison to other households in the village (i.e. 1 = worse than others, 2 = same as others, 3 = better than others). Participants were also asked to report on other adults and children living in the household by age and sex and how many meals they ate each day as a measure of food security.

### Self-report PTSD symptoms, anxiety, and depression

The exposure to the traumatic events section of the questionnaire was adapted from the Harvard Trauma Questionnaire (HTQ), a multipart cross-culturally validated instrument that measures traumatic events and PTSD (Mollica *et al*., 1992; Kohli *et al*., 2013). Exposure to trauma was analyzed as a continuous variable (0–18 different traumatic events). A 16-item version of section 4 of the HTQ was used to identify symptoms consistent with PTSD in the past 7 days (Mollica *et al*., 1992; Kohli *et al*., 2013). The depression and anxiety components of the Hopkins Symptom Checklist (HSCL) were used for reporting the experience of symptoms that bothered or distressed the respondent during the past 1 month. The HTQ and HSCL have been used widely in conflict-affected and humanitarian emergencies, and both have strong psychometric properties for measuring of traumatic events and symptoms consistent with PTSD and depression in conflict-affected settings (Mollica *et al*., 1993; Onyut *et al*., 2009; Roberts *et al*., 2009). In this sample, Cronbach's *α* was 0.86 for anxiety, 0.85 for depression, and 0.89 for PTSD. The research team had previously used the HTQ and HCSL in studies conducted in DRC (Kohli *et al*., 2013).

### Self-report sleep disturbance and interference

Participants completed the PROMIS short form v1.0-Sleep Disturbance 8a and the PROMIS short form Sleep-Related Impairment 8a Interference (Yu *et al*., 2011). These surveys were translated and back-translated in French and local languages (Swahili and Mashi), pilot-tested with skilled Congolese research assistants on tablet computers. The Sleep Disturbance 8a form measures self-reported difficulties falling asleep and staying asleep, as well as sleep satisfaction while the sleep-related impairment questionnaire measures self-reported alertness, sleepiness, tiredness, and functional impairments associated with sleep problems during waking hours within the past 7 days. Both measures use a five-point Likert scale, and responses on each scale are summed. The minimum and maximum scores on each questionnaire are 8 and 40, respectively, with higher scores representing higher severity of sleep impairment and sleep interference. Raw scores were converted to *T* scores according to the scoring manual (HealthMeasures, 2020). All participants completed the survey at the end of the study when the AW-S were collected.

### Self-report health and physical function

Given that pain, energy level, and sleep disruption are closely related symptoms that frequently cluster together and are prominent in PTSD (Benedict *et al*., 2020), measures of bodily pain and energy level that were collected at baseline in the parent study were incorporated into the current analysis. Participants were asked to rate on a 1–4 scale of how much bodily pain they have had in the last 4 weeks, where 1 = none, 2 = very mild, 3 = moderate, 4 = severe, and 5 = very severe. The energy level was assessed by asking participants how much energy they have had in the past 4 weeks on a 1–4 scale, where 1 = none, 2 = a little, 3 = quite a bit, and 4 = very much. Overall health was measured with one item, rating health from poor to excellent in the past 30 days. Participants were asked to rate how much physical health limited their physical activities and how much difficulty they had doing their daily work on a 1–4 scale where 1 = not at all, 2 = very little, 3 = quite a lot, and 4 = could not do physical activities.

### Habitual physical activity and sleep assessment by actigraphy

Activity data were collected during 60 s epochs for the duration of the study. The AW-S contains a sensitive omnidirectional accelerometer that counts wrist movements to estimate activity and sleep duration (Gironda *et al*., 2007), and wrist actigraphy has been validated against PSG and demonstrated a total sleep duration correlation of >0.9 (Lichstein *et al*., 2006). Sleep and activity measures were calculated with the Actiware 5.0 software package (Respironics Inc. Bend, OR, USA) using the software's ‘medium’ sensitivity setting, and the default sleep onset and sleep end defined by ten consecutive immobile minutes, and ten consecutive minutes of activity, respectively. Nighttime sleep parameters including total sleep time (TST), sleep onset latency (SOL; the time required for a full transition from wakefulness to sleep), sleep efficiency (SE; the total time spent asleep/total time attempting to sleep), and wake after sleep onset (WASO; time spent awake after falling asleep but before final awakening). Intra-individual activity in TST, SOL, SE, and WASO, was examined across each night of data collection by calculating the root mean squared successive difference (rMSSD). Calculation of rMSSD has been used in prior studies to examined intra-individual variability (IIV) in sleep parameters and PTSD symptomatology (Straus *et al*., 2015; Mellman, 2018). The rMSSD is obtained by first calculating the difference between successive observations (i.e. the difference between the SE on days 1 and 2, days 2 and 3 etc.), which is then squared. Next, the square root of the average of these squared values is calculated. Daytime actigraphy data were used to calculate the number of minutes of daytime napping using the same criterion, i.e. 10 min of continuous inactivity. In addition, activity data were used to calculate habitual physical activity while awake, and peak physical activity level across the days of assessment.

### Data analysis

Frequency distributions and descriptive statistics were examined for all study variables. Summary statistics of demographic data were calculated as mean (s.d.) for continuous variables and as percentages for categorical variables. Non-normally distributed variables were log-transformed before analysis. Changes in self-report sleep disturbance and interference during the study were examined using paired *t* tests. Multivariate linear regression models were used to examine the relationship between variables. All statistical tests were performed using SPSS version 26.0 (IBM Corporation). We used Bonferroni correction to adjust for multiple predictors using the following formula: 0.05/number of predictors.

## Results

### Characteristics of the sample

Table 1 summarizes responses to study questionnaires collected from 30 participants recruited into the study (7 men and 23 women). The majority of participants were between 45 and 60 years of age (43.3%), married (57%), and had young children (0–5 years) living at home (53%). Over half believed that they were worse off economically than their neighbors, and 33% reported eating only one meal per day while the majority (60%) ate two meals per day. Over 60% of the sample reported moderate to severe bodily pain, and no or little energy. Over 50% rated their overall health in the last 4 weeks as fair or poor. The mean scores on the anxiety and depression subscales of the HSCL were 1.8 ± 0.5, respectively. Twenty-three percent of the sample scored ⩾2.5 on the HTQ, which is indicative of a diagnosis of PTSD.

**Table 1.** Participant characteristics (*N* = 30)

Characteristic	% or mean ± s.d.
Female	76.7
Age	
20–34	30
35–44	10
45–60	43.3
>60	16.7
Marital status	
Married	57.1
Widowed	35.7
Abandoned	7.1
Children in household	
0–5 years	53.6
6–17 years	78.6
Perceived household wealth	
Worse off	55.6
Same	44.4
Meals per day	
1	33.3
2	60.0
3	6.7
Bodily pain	63
None or very mild	37
Moderate	33
Severe	30
Energy level	
None or a little	63.3
Quite a lot or very much	36.7
Overall health	
Very good or good	46.7
Fair or Poor	53.3
HSCL	
Anxiety subscale (range 1–4)	1.8 ± 0.5
Depression subscale (range 1–4)	1.8 ± 0.5
HTQ	
Trauma symptoms (range 1–4)	1.9 ± 0.5
Has PTSD (⩾ 2.5)	23

Note: Data represent means and s.d. or percent of the sample. HSCL is the Hopkins Symptom Checklist, HTQ is the Harvard Trauma Questionnaire.

### Feasibility and acceptability of objective activity and sleep assessment

All 30 participants approached by the study staff gave verbal consent to participate in the study and underwent training on how to wear the AW-S. Among the 30 participants, a few issues were raised by participants during informed consent, training, and follow-up discussion. Participants raised concerns about consistently wearing the AW-S; specifically, they raised the concern the AW-S would record their sexual activity if worn during the night, and the possibility of jealousy from others in the village if they saw them wearing the AW-S. The PFP staff worked with each participant to help them feel comfortable with the confidentiality of data and strategies to cover the watch (wear long sleeves) when outside the home. During follow-up visits the next day, two female participants decided to withdraw from the study because of concerns raised by male family members about wearing the AW-S. Of the remaining 28 participants, 23 wore the AW-S as instructed, only taking off the device during periods when coming into contact with water. Five participants started to wear the AW-S from Tuesday morning instead of Monday before sleep but wore the device for the remainder of the study period. For these five participants, we collected sleep and awake activity data across 4 nights and 4 days. For the remaining 23 participants, we collected sleep and awake activity across 5 nights and 4 days. All participants who wore the device marked sleep and wake cycles and periods of non-wear using the marker. After participation, some participants reported that they slept much better while wearing the device, believing the AW-S was a treatment to improve sleep.

### Self-report and objectively measured sleep quality

Table 2 shows self-report sleep disturbance and interference, mean and IIV in sleep parameters, daytime napping, and habitual physical activity data across the 4–5 nights and 4 days of the study, respectively. On average, participants fell asleep within 30 min of going to bed, and slept 6–7 h each night, with an average SE of 78 ± 10%. Participants napped for a little over an hour a day (73.3 ± 68.2 min.) IIV in nighttime sleep parameters across the 4–5 nights of assessment was highly variable (Table 2). Mean (s.d.) self-report sleep disturbance and sleep interference *T* scores were 51.9 ± 8.0 and 47.5 ± 8.7, respectively (Table 2).

**Table 2.** Intra-individual variability in sleep and physical activity parameters across days

Parameter	Mean (s.d.)	Range
Sleep parameters across all days		
Bedtime	8:54 PM	8:00 AM–12:08 PM
Rise time	5:37 AM	3:55 AM −6:33 AM
Night TST (h)	6.7 (1.0)	3.6–8.9
Onset latency (min)	15.3 (14.7)	0.3–56.2
SE (%)	78.0 (10.8)	46.6–93.1
WASO (min)	62.3 (25.0)	18.5–175.2
Day TST (min)	73.3 (68.2)	10.4–269.8
Intra-individual variability in sleep parameters^b^		
Night TST (min)	22.5 (26.6)	0.3 −109.4
Onset latency (min)	8.7 (5.9)	1.8 −26.9
SE (%)	65.6 (30.6)	25.7–154.0
WASO-variability (min)	33.5 (19.5)	4.8–97.6
Self-report sleep outcomes		
Sleep disturbance	51.9 (8.0)	28.9–63.7
Sleep interference	47.5 (8.7)	30–61.3
Change sleep disturbance	−9.2 (14.8)*	−37.2–12.5
Change sleep interference	−12.4 (13.7)**	−35.5–5.9
Habitual physical activity across all days		
Average daily activity (cpm)	607 (295)	142–1495
Peak daytime activity (cpm)	2112 (609)	721–3417

Note: Data represent minimum, maximum, means and s.d. or percent of the sample.

Abbreviations: TST is total sleep time, WASO is wake after sleep onset, and SE is sleep efficiency. In the table please remove [b] from intra-individual variability in sleep parameters and change WASO-variability to WASO in this section as well.

### The relationship between self-report and objectively measured sleep parameters and trauma symptoms

We found no significant association between self-report sleep disturbance and interference and objectively measured sleep parameters (all *p* > 0.3, data not shown). To examine the relationship between self-report and objectively measured sleep parameters and trauma symptoms, we performed linear regression models with the dependent variables of self-report sleep disturbance and interference, average and IIV-TST, -SE, -SOL, or -WASO, and the independent variables of scores on the HSCL anxiety and depression subscales and the HTQ trauma symptom subscale. Because age and level of bodily pain could influence sleep, we included these variables in the model. Table 3 shows a summary of the linear models of predictors for each self-report and objectively measured sleep parameter. There was no statistically significant association between any objectively measured sleep parameters and predictor variables (*p* > 0.01, Table 3) except for the level of bodily pain; higher levels of pain were associated with longer sleep time (*β* = 0.63, *p* = 0.003, adjusted *R*^2^ = 0.279). The same analyses were performed for day time napping. Again we found no statistically significant relationship between daytime napping and the predictor variables (Table 3).

**Table 3.** Linear model of predictors of objectively measured sleep parameters

	Anxiety^a^			Depression^b^		Trauma symptoms^c^		Age		Bodily pain	
Sleep parameter	Adj. *R*^2^	*β* (95% CI)	*p*	*β* (95% CI)	*p*	*β* (95% CI)	*p*	*β* (95% CI)	*p*	*β* (95% CI)	*p*
Disturbance	−0.003	0.24 (−6.2, 12.3)	0.497	−0.54 (−14.8, 1.0)	0.080	0.003 (−9.7, 9.8)	0.994	−0.16 (−3.4, 1.8)	0.504	−0.11 (−3.3, 2.1)	0.652
Interference	0.153	0.64 (−0.2, 12.7)	0.056	−0.26 (−8.2, 2.8)	0.322	−0.19 (−8.8, 5.1)	0.577	−0.36 (−3.3, 0.37)	0.111	−0.09 (−2.4, 1.6)	0.687
TST	0.279*	−0.49 (−2.2, 0.2)	0.109	0.34 (−0.3, 1.7)	0.180	0.09 (−1.1, 1.4)	0.765	0.05 (−0.3, 0.3)	0.787	0.63 (0.2, 0.8)	0.003
TST-IIV	0.272**	0.26 (−0.9, 2.2)	0.383	−0.34 (−2.2, 0.4)	0.175	0.44 (−0.5, 2.7)	0.163	0.40 (0.03, 0.8)	0.036	0.28 (−0.1, 0.7)	0.151
SE	0.102	−0.20 (−15.7, 8.5)	0.544	0.51 (−1.1, 19.4)	0.076	−0.36 (−19.1, 6.1)	0.292	−0.10 (−3.6, 2.2)	0.613	0.35 (−0.6, 6.1)	0.107
SE-IIV	0.053	0.73 (0.03, 1.2)	0.040	−0.21 (−0.6, 0.3)	0.449	−0.23 (−0.8, 0.4)	0.519	−0.12 (−0.2, 0.1)	0.563	−0.16 (−0.2, 0.1)	0.453
WASO	−0.050	−0.14 (−0.8, 0.5)	0.685	−0.48 (−1.1, 0.13)	0.116	0.42 (−0.3, 1.1)	0.264	−0.06 (−0.2, 0.2)	0.798	−0.002 (−0.2, 0.2)	0.994
WASO-IIV	0.121	−0.14 (−1.1, 0.7)	0.669	−0.58 (−1.5, −0.03)	0.042	0.62 (−0.1, 1.7)	0.080	−0.11 (−0.3, 0.2)	0.574	0.40 (−0.02, 0.4)	0.066
LOS	0.223	0.18 (−1.2, 2.1)	0.561	−0.37 (−2.4, 0.4)	0.161	0.43 (−0.5, 2.8)	0.186	0.37 (−0.01, 0.7)	0.056	0.33 (−0.1, 0.8)	0.099
LOS-IIV	−0.056	0.30 (−0.6, 1.4)	0.406	−0.51 (−1.5, 1.5)	0.100	0.19 (−0.7, 1.3)	0.607	−0.03 (−0.3, 0.2)	0.890	0.08 (−0.2, 0.3)	0.707
Napping	0.146	−0.15 (−1.3, 0.9)	0.653	0.35 (−0.35, 1.5)	0.204	−0.34 (−1.8, 0.5)	0.308	0.38 (−0.02, 0.6)	0.067	0.08 (−0.2, 0.3)	0.671

Note: Independent variables included in the model are scores on the HSCL anxiety^a^ and depression^b^ subscales and the HTQ trauma symptom subscale^c^, age, and level of bodily pain. Separate models were used to evaluate each sleep parameter. Data are presented as standardized Beta coefficients (*β*), 95% confidence interval (95% CI), and *p* value. Statistical significance set to *p* ⩽ 0.01 to adjust for multiple predictors in the model. Abbreviations: TST is total sleep time (h), SE is sleep efficiency (%), WASO is wake after sleep onset (min), SOL is sleep onset latency (min), IIV is intra-individual variability. *p = 0.033, **p = 0.036

### The relationship between objectively measured sleep parameters and functional outcomes

To examine the relationship between objectively measured sleep parameters and functional outcomes, we performed linear regression with the dependent variables of peak daily physical activity, limitations of health on physical activities, and difficulty doing daily work in separate models. The independent variables were average TST, age, and bodily pain, as above, and energy level, which we reasoned that like age and pain, would impact functional outcomes. There was no statistically significant association between peak daily physical activity and any of the predictors (*p* < 0.01, Table 4). There was no statistically significant association between the ability to perform daily work or limitations of health on physical activities and any of the predictor variables (*p* > 0.0125) except for bodily pain. Those with higher levels of bodily pain were more likely to report that their physical health limited their physical activities (*β* = 0.85, *p* < 0.0001, adjusted *R*^2^ = 0.634) and had greater difficulty doing daily work (*β* = 0.91, *p* < 0.0001, adjusted *R*^2^ = 0.645).

**Table 4.** Linear model of predictors of functional outcomes

Functional Outcome	TST			Age		Bodily Pain		Energy	
	Adj. *R*^2^	*β* (95% CI)	*p*	*β* (95% CI)	*p*	*β* (95% CI)	*p*	*β* (95% CI)	*p*
Peak daily activity	0.153	−0.24 (−386, 115)	0.275	−0.07 (193, 136)	0.722	−0.44 (−406, 0.3)	0.050	−0.15 (−431, 216)	0.499
Health limitations	0.634*	−0.04 (−0.3, 0.2)	0.807	−0.2 (−0.3, 0.1)	0.164	0.85 (0.4, 0.8)	0.000	0.04 (−0.3, 0.4)	0.773
Work difficulty	0.645*	0.03 (−0.2, 0.2)	0.814	−0.07 (−0.2, 0.1)	0.578	0.912	0.000	0.27 (−0.03, 0.5)	0.074

Note: Independent variables included in the model are TST, age, level of bodily pain, and energy level. Separate models were used to evaluate each functional outcome. Dependent variables were peak daily activity from actigraphy, Data are presented as standardized Beta coefficients (*β*), 95% confidence intervals (95% CI) and *p* value. Statistical significance was set to *p* ⩽ 0.0125 to adjust for multiple predictors in the model. Abbreviations: Health limitations and work difficulty represent how much physical health limited physical activities and how much difficulty respondents had to do their daily work with higher scores representing greater limitations and difficulty. TST is total sleep time (h). *p < 0.0001

## Discussion

This study provides important contributions to our knowledge in two important ways. First, the use of actigraphy to collect objective measures of daily physical activity and sleep quality in men and women in rural post-conflict settings is feasible and acceptable. Second, our preliminary findings suggest that sleep quality and physical function in men and women survivors of prolonged conflict are not associated with the self-report of adverse events or mental health symptoms, but rather bodily pain. There have been a handful of studies that have objectively measured sleep parameters using wrist-worn actigraphy in countries with limited resources (Knutson, 2014; de la Iglesia *et al*., 2015; Yetish *et al*., 2015; Samson *et al*., 2017). These studies focused in part on comparing sleep quality in resource-poor communities without electric lighting with industrialized countries like the United States (US). Similar to these studies, we found that participants slept on an average <7 h per day, going to bed ~3 h after sundown and getting out of bed at sunrise. In the US, a short sleep duration, defined as <7 h of sleep per 24 h period, occurs in 24–50% of the population depending on the state and is associated with poor health outcomes including cardiovascular disease, diabetes, and depression (Anothaisintawee *et al*., 2016; Kwok *et al*., 2018; Lo *et al*., 2018). Poor sleep quality is a significant barrier to functioning in the household and community, limiting activity, productivity and socialization (Grandner, 2017).

Of note, 68% of our sample had poor sleep in that they slept <7 h per night. One-third of the participants met the criteria for PTSD, which is higher than WHO estimates of the prevalence of mental disorders (depression, anxiety, PTSD, bipolar disorder, and schizophrenia) as 22⋅1% (95% UI 18⋅8–25⋅7) at any point in time in the conflict-affected populations assessed (Charlson *et al*., 2019). However, symptoms of PTSD, anxiety, or depression did not significantly influence sleep quality, which suggests other factors contribute to sleep quality in this setting. Factors influencing sleep quality in prior studies set in resource-poor settings included daily and seasonal temperature fluctuations, length of daylight, and sleeping situation. Knutson *et al*., who examined sleep quality in a community without electricity in Haiti, found that household size was negatively associated with WASO (Knutson, 2014), meaning more significant sleep disruption in larger households. In this study, we found no association between the number of household members and sleep quality, although we lacked detail on the precise sleeping arrangements such as the number of people in the sleep setting. Yetish and colleagues examined the impact of seasonal changes in light and temperature on sleep in three different pre-industrial communities and found that individuals slept longer in the winter months likely due to cooler climate and longer dark periods (Yetish *et al*., 2015). The villages from which participants were recruited for this study lie within 40 km (~25 miles) from Bukavu, DRC. Bukavu lies 175 miles north of the equator, and as a result, there is little seasonal variation in light and dark (sunrise at 6:00 AM and sunset at 6:00 PM) or temperature (high of 75–79 °F, low 55–59 °F). It is possible that cooler temperatures during the night could have impacted participants sleep quality in this study if the bedding was not sufficient to remain warm.

One-third of the participants in this study ate only one meal a day; an indication of food insecurity. Food insecurity defined by the United Nations as limited access to a diet of sufficient nutritional quality and quantity has been linked to poor health outcomes including poor sleep (Ding *et al*., 2015; Jordan *et al*., 2016; Arenas *et al*., 2019). In a recent systematic review and meta analysis adults in the US with food insecurity had increased anxiety odds ratio (OR = 2.41), depression (OR  = 2.71), and sleep disorders (OR  = 1.81) (Arenas *et al*., 2019). Although we did not observe a relationship between food security, mood, sleep quality, and functional outcomes including daily work, the inclusion of a more extensive food security measure should be included in future studies. In the sleep study, we used how many meals are eaten per day as a measure of food security. In future studies, we will include measures such as the household dietary diversity scale (HDDS) that assesses the total number of food groups (range: 0–12 items) consumed by the household members in the previous day and night (Swindale and Bilinsky, 2006).

Of interest, several participants reported that they slept much better while wearing the AW-S, which may explain the lack of the association between self-reported and objectively measured sleep quality. Other studies have found only a weak-moderate association between self-report and objectively measured sleep parameters (Jackson *et al*., 2018), with people often over-reporting sleep difficulties. In addition, despite one-third of the participants meeting the criteria for PTSD we did not see any association between self-report or objectively measured sleep outcomes and trauma-related symptoms including anxiety, depression, or mean trauma scale.

By far, the biggest contributor to sleep quality, and functional outcomes including peak daily physical activity, and ability to work was the level of bodily pain. We found that individuals with greater bodily pain slept significantly longer at night than those with milder pain. One might expect that more severe bodily pain would disrupt sleep leading to shorter sleep times but this was not the case in this study. Greater bodily pain was associated with greater physical limitations and more difficulty working. All study participants were engaged in small scale agriculture and animal husbandry which requires daily strenuous physical activity and manual labor (lifting and carrying heavy loads over distances, bending, kneeling, and squatting). Thus, both men and women in the study are at risk for agricultural work-related musculoskeletal (MSK) pain, often reported as low-back pain, knee pain, shoulder and neck pain (Naidoo *et al*., 2009; El-Sayed *et al*., 2010; Min *et al*., 2016; Morris *et al*., 2018). Agricultural work-related MSK pain may lead to further negative health and economic consequences such as reduced sleep quality resulting in lower daily activity and productivity leading to reduced income and increased stress and symptoms of poor mental health including depression and anxiety because of not being able to meet household basic needs.

## Study limitations

The study sample is small and focused in a few villages in one territory in the South Kivu region of Eastern DRC, therefore, the findings are not generalizable to other settings. However, the sample of 30 participants is acceptable for a feasibility and acceptability study (Arain *et al*., 2010). Future studies would be strengthened by including validated measures to better characterize pain associated with occupational exposure (Naidoo *et al*., 2009; El-Sayed *et al*., 2010; Min *et al*., 2016; Morris *et al*., 2018). This information is useful for future economic empowerment programs in rural communities as outcomes may be improved by developing locally relevant strategies for occupational health, specifically preventing injuries and chronic pain with small-scale farmers. At the time of analysis, information on drug and alcohol use had only been collected for male participants in the parent study. Given the impact that drug and alcohol use could have on sleep quality, mood, and functional outcomes examined in this study, assessment of recreational drug and alcohol use is warranted in follow-up studies (Colrain *et al*., 2014). Another potential limitation of this study is the time frame during which sleep data were collected for the analysis of IIV in sleep outcomes. While a minimum of 3 days is recommended for the assessment of daily sleep parameters, longer periods may be recommended for the assessment of IIV (Aili *et al*., 2017).

## Conclusions

This initial study supports the need for continued research related to sleep, bodily pain and mental health in humanitarian settings (Murray *et al*., 2014). There is a significant research focus and increased interventions to address self-reported mental health and exposure to conflict, however, limited research exists on use of technology to collect objective measures associated with health, thus limiting the focus in occupational health and preventing and responding to injuries and chronic pain associated with a lifetime of manual labor.
